# Gluten-Free Diet: Gaps and Needs for a Healthier Diet

**DOI:** 10.3390/nu11010170

**Published:** 2019-01-15

**Authors:** Valentina Melini, Francesca Melini

**Affiliations:** CREA Research Centre for Food and Nutrition; Via Ardeatina 546, I-00178 Rome, Italy; valentina.melini@crea.gov.it

**Keywords:** coeliac disease, gluten-free diet, nutritional adequacy, gluten-free diet co-morbidities, obesity, gut microbiota, metabolic syndrome

## Abstract

The gluten-free diet (GFD) is currently the only effective treatment in remitting the symptoms of coeliac disease (CD), a chronic systemic autoimmune disorder caused by a permanent intolerance to gluten proteins in genetically susceptible individuals. The diet entails the substitution of gluten-containing products with gluten-free-rendered products. However, over recent decades the nutritional profile of gluten-free (GF) food products has been increasingly questioned within the scientific community. The aim of this paper is to review the nutritional profile of gluten-free-rendered products currently available on the market, and discuss the possible relationship thereof with the nutritional status of coeliac patients on a GFD. Key inadequacies of currently available GF products are low protein content and a high fat and salt content. More adequate levels of dietary fiber and sugar than in the past have been reported. Population studies confirmed the above mentioned inadequacies. Further efforts are required to conceive adoptable interventions for product development and reformulation in order to achieve compliance with nutritional recommendations.

## 1. Introduction

According to the World Health Organization (WHO), a diet is healthy when it contributes to protecting against malnutrition in all its forms: under- and over-nutrition [1]. A healthy diet also protects from non-communicable diseases (NCDs), such as diabetes, heart disease and stroke, which are a major issue for public health and a burden for health care systems in Western countries.

The essential feature for a diet to be healthy is being varied and balanced, as outlined in most worldwide dietary guidelines. It must be high in fruits and vegetables and rich in whole grains, low-fat or non-fat dairies, fish, legumes, and nuts, but low in refined grains [1]. It must assure a high intake of dietary fiber (DF) and polyunsaturated fatty acids, and a low intake of fat, sugars, salt and saturated fatty acids.

Food intolerances and allergies, and some medical conditions require “special diets” to keep people healthy. The gluten-free diet, DASH (Dietary Approaches to Stop Hypertension) diet, healthy kidney diet, ketogenic diet and low-FODMAP (Fermentable Oligo-, Di-, Mono-saccharides And Polyols) diet are some examples of diets followed for medical reasons. All these diets share the limitation or exclusion of specific food components or categories that might trigger the allergy/intolerance or be harmful for some subjects.

A gluten-free diet (GFD) requires the complete exclusion of gluten, a protein complex present in food products from wheat, rye, barley, oats, spelt, kamut or their hybridized strains. It comprises only naturally gluten-free (GF) food products (e.g., legumes, fruit and vegetables, unprocessed meat, fish, eggs and dairy products) and/or substitutes of wheat-based foods, specially manufactured without gluten or having a gluten content lower than 20 ppm, as per European legislation [2,3].

Three conditions require treatment with GFD: Wheat allergy, non-coeliac gluten-sensitivity, and coeliac disease (CD). Wheat allergy is an immunologic reaction to wheat proteins especially common among children [4]; non-coeliac gluten sensitivity is the disorder that individuals may show upon ingestion of the above-mentioned cereal proteins, with improvements when these are removed from diet [4]; and CD is a chronic, small-intestinal immune-mediated enteropathy characterized by specific antibodies against tissue transglutaminase 2 (anti-TG2), endomysium, and/or deamidated gliadin peptide, affecting one in every hundred individuals in Western population [5]. Long-term risks associated with CD, such as lymphoma, osteoporosis and anemia, have been reported [6,7,8].

A strict adherence to the GFD and a lifelong exclusion of gluten from the diet is the first-line treatment and is currently the only effective therapy for CD. Although conceptually simple, the observance of a GFD is difficult for the impact it may have on life quality; this depends upon environmental and individual factors. The availability of GF foods, their value for money, and clear labelling are among the main factors affecting dietary compliance. The availability of GF products has dramatically grown over the past five years: GF products can be purchased in major supermarkets, health food stores and online shops [9]; however, they remain significantly more expensive than gluten-containing food products [10,11,12,13,14]. As a component of some grains, gluten is typically not listed separately in the product label, hence foods that contain gluten might be difficult to identify. Moreover, it may be present as a hidden food component. Due to its technological properties, it is used as a flavor enhancer, thickener, emulsifier, filler and fortifier, and might be hidden under the terms “flavorings”, or “hydrolyzed vegetable proteins”. Impairment in social leisure activities of CD patients has been also observed [15].

The aim of this paper is to review the nutritional profile of the gluten-free-rendered products currently available on the market and discuss the possible relationship thereof with the nutritional status of coeliac patients on a GFD. The paper then provides an overview of nutrient inadequacies at CD diagnosis and during GFD adherence. Following this, the nutritional composition of currently available GF wheat-substitutes, which emerged from food surveys conducted over the last five years, is explored and the most recurrent inadequate nutrient uptakes pinpointed. Based on the above, the risk of co-morbidity occurrence during GFD adherence and changes in gut composition are finally presented.

## 2. Materials and Methods

### 2.1. Literature Search

The study layout was first designed by the authors. An extensive literature search for papers on the planned topics was then conducted on PubMed and SCOPUS databases. Time limits were set, so as to obtain only papers published over the last five years. Authors also screened the reference list of eligible papers. Several combinations of terms were used, depending on the following aspects: Nutritional inadequacies at CD diagnosis and during adherence to a GFD, nutritional profile of GF bakery products, and risk of co-morbidity diseases upon adherence to GFD. The same keywords were used to perform the query in both aforementioned literature databases. The MeSH terms are specifically reported in Table 1.

### 2.2. Including and Excluding Criteria

**Figure 1:**
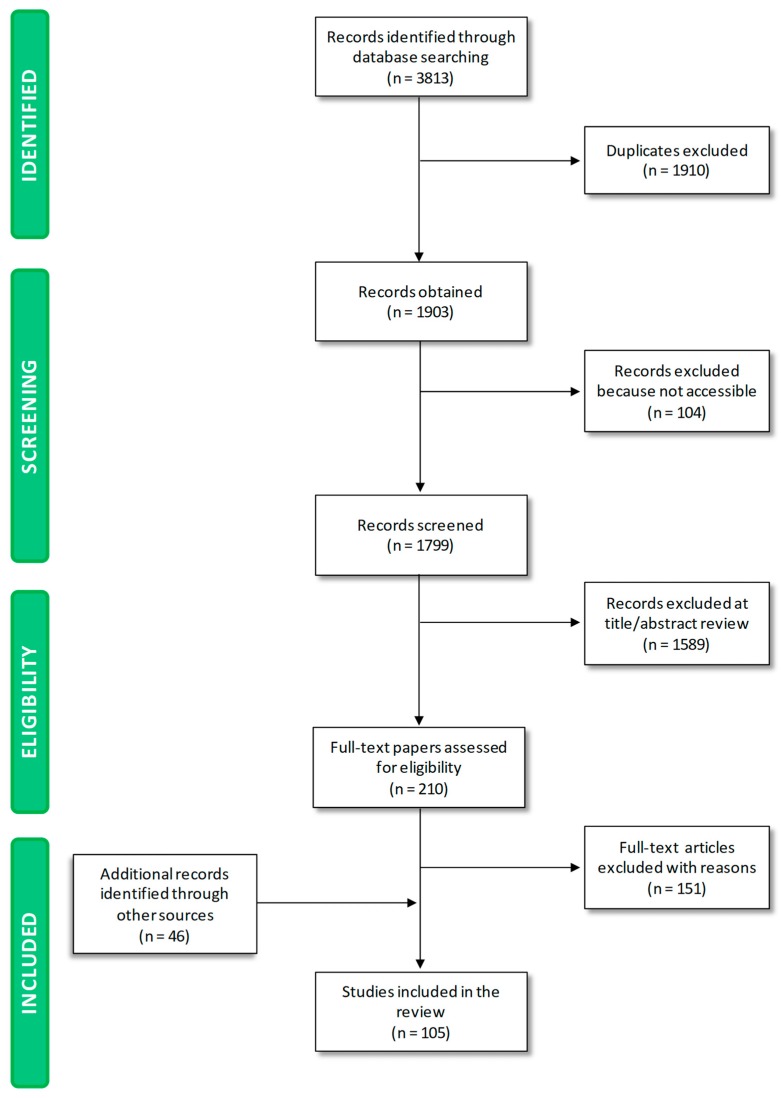
PRISMA (Preferred Reporting Items for Systematic Reviews and Meta-Analyses) flow diagram.

As shown in Figure 1, the initial process yielded 3813 publications. In total, 1910 papers were excluded because they were duplicates. A total of 104 papers were not accessible to the authors, and 1589 were excluded during title/abstract review because they dealt with aspects falling beyond the scope of the paper.

This procedure resulted in 210 potentially relevant papers. A total of 151 papers were excluded on the basis of the full-text screening. Upon screening of reference lists of eligible papers and consultation of health/regulatory organization websites, 46 studies were included, because they were relevant to the topics of the manuscript. A total of 105 papers were finally selected. The key information contained within was reviewed, extracted and considered for analysis.

### 2.3. Software

Literature search data were collected on Microsoft Office Excel 2010 spreadsheets. The software MATLAB 8.2 (MathWorks Inc., Natick, MA, USA) was used to identify duplicates.

## 3. Results and Discussion

### 3.1. Nutritional Status of CD Patients at Diagnosis

The nutritional status of CD patients at diagnosis depends on the length of time the disease is active, the extent of intestinal inflammation, the degree of malabsorption, and dietary intake [16]. Malabsorption, resulting from the villous abnormalities in the small intestine, leads to multiple nutritional deficiencies.

**Figure 2:**
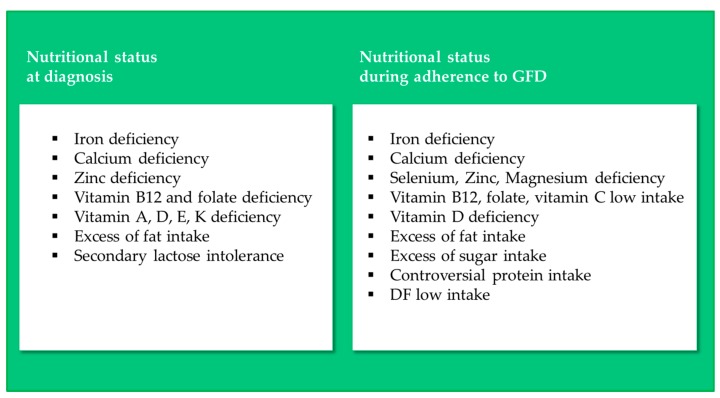
Nutritional inadequacies in CD patients at diagnosis and during adherence to a GFD.

Deficiencies in iron, calcium, zinc, vitamin B_12_, vitamin D and folate are by far the most common nutritional inadequacies claimed for newly-diagnosed coeliac patients, as tested by analysis of blood samples [17,18,19,20,21,22] (Figure 2).

Iron-deficiency anemia is one of the most recurrent extra-intestinal manifestations of CD and has been detected in almost 46% of subclinical CD cases [17]. The main cause relies on the fact that the villous atrophy is primarily located in the duodenum, which is also the main site of iron absorption.

Calcium deficiency and subsequent metabolic bone diseases are also a frequent co-morbidity in CD patients [18]. Approximately 75% of untreated adult coeliac patients suffer from low bone mineral density, as calcium and vitamin D are absorbed in the duodenum [17]. In young patients (i.e., children and adolescents), calcium deficiency may cause growth problems and difficulties in peak bone mass achievement, whereas in elderly people it results in a lowered mineral density and increased bone fracture risks [18]. Osteopenia and osteoporosis are considered signs of atypical CD presentation [19].

Zinc deficiency is claimed to be a consequence of increased endogenous loss of that mineral, rather than an abnormal zinc absorption [20]. It is known that the gastrointestinal tract is crucial for the homeostatic control of zinc, and involves a complex interplay of host, dietary and environmental factors [23]. Due to its role in several reactions and biochemical functions, a zinc deficiency can affect protein synthesis and leads to growth arrest [24].

As regards vitamins, vitamin B_12_ deficiency is found in 8%–41% of newly-diagnosed coeliac people, as its absorption mainly occurs in the ileum [17,20]. According to some authors, the reasons of that deficiency are nevertheless not well-known, and some scientists claim they may also relate to small intestinal bacterial overgrowth which often occurs as a complication of the small intestinal injury [16]. Prevalence of folate deficiency in untreated coeliac people ranges from 18% to 90%, depending on the technical measurement of folate in comparison with folic acid [17,21,22]. Deficiencies of the fat soluble vitamins A, D, E and K in untreated CD patients have also been reported [20], and vitamin D deficiency has been specifically associated to osteomalacia.

Macronutrient inadequacies are rarely identified at diagnosis. Among other nutrient inadequacies, Kupper [25] reported protein deficiency at diagnosis. Coeliac disease is, in fact, reported among causes of protein-losing enteropathy [26]. Shepherd and Gibson [27] compared newly-diagnosed, untreated patients to long-term treated CD people in their study, and stressed an excess of fat intake (specifically, saturated fats) in both men and women at CD diagnosis.

Secondary lactose intolerance resulting from decreased lactase production by the damaged villi is also common [17,28].

### 3.2. Nutritional Status of CD Patients Adhering to a GFD

The substitution of gluten-containing (GC) food products with GF ones in the diet, and the following recovery of mucosal functionality in CD patients, can be assumed to change the nutritional status of patients observed at diagnosis (Figure 2). Several population studies have investigated the nutritional status of CD patients adhering to a GFD.

Food records and questionnaires have been used to evaluate the adherence to the GFD and to calculate nutrient intake. Data regarding nutrient intake have been compared to values recommended by dietary guidelines. Moreover, healthy subjects, consuming GC food products, have been used as controls.

As far as macronutrient intake is concerned, several studies performed on children, adolescents and/or adults agree in reporting GFD as an unbalanced diet. Fat intake is commonly higher than recommended [29,30,31,32,33,34,35,36]. However, disagreement among studies emerged when fat intake of CD subjects adhering to GFD were compared to controls. Mariani et al. observed that lipid consumption was higher in CD adolescents than in controls [36]; according to Hopman et al., fat intake was comparable with the general population [34]; while Zuccotti et al. reported a lower fat intake in CD patients than in controls [37]. This divergence among results might be due to differences in dietary habits of the healthy subjects used as controls from country to country, and to the variability of GF product formulations from brand to brand. As a matter of fact, two of the above mentioned studies were performed in Italy and one in Germany. No information is available about the season during which data were collected; however, it should not be neglected that during winter, foods with a higher fat content are generally preferred. Moreover, the study of Mariani et al. was performed in 1998, the study of Hopman et al. in 2006, and Zuccotti et al. in 2013: It is likely that changes in GF product formulation occurred from 1998 to 2013.

Controversial findings have been claimed for protein intake. At the end of last millennium, Mariani et al. observed that protein intake in coeliac patients was high [36]. More recently, Shepherd and Gibson [27] found that in a female study population, the mean intake of protein post-diagnosis was significantly lower after 12 months on the GFD. Van Hees et al. [38] observed that coeliac patients on a long-term GFD consume significantly less vegetable protein than healthy controls.

As regards carbohydrates, population studies generally report a higher intake of sugars in CD than in controls [31,39], and all agree on a low intake of DF [29,32,33,34,36,40,41] in CD subjects adhering to GFD.

Population studies highlighted that the GFD was found ineffective in resolving the mineral and vitamin deficiencies observed at diagnosis. Iron deficiencies were reported in CD subjects adhering to a GFD by Mariani et al. [36], Thompson et al. [42], Martin et al. [32], Sue et al. [29], and Shepherd and Gibson [27]. In contrast, Thompson et al. [42] specifically observed that all male CD patients and 44% of CD women belonging to the study population consumed the daily recommended amount of iron. However, Öhlund et al. [33] found that iron and also calcium intake was higher in CD children than in controls.

Calcium deficiencies were extensively reported [18,25,27,29,36,42,43] in CD patients adhering to a GFD. In contrast, the study by Öhlund et al. [33] reported a nutrient density of calcium higher in CD children than in controls. 

The intakes of selenium, zinc and magnesium were found to be lower in CD subjects than in controls [27,33].

As regards vitamins, deficiencies were also observed in the GFD, with vitamins B_12_, folate and vitamin D being the most deficient. Hallert et al. [44] observed a lower intake of vitamin B_12_ and folate in half of the studied coeliac patients on a GFD for 10 years compared to controls. This poor vitamin status was confirmed by the evaluation of total plasma homocysteine. This status could have clinical implications considering the linkage between vitamin deficiency, elevated total plasma homocysteine levels and cardiovascular disease. A lower intake of vitamin B_12_, along with folic acid and vitamin C, was also reported by Martin et al. [32].

Vitamin D deficiency has also been observed in CD subjects on a GFD [29,43,45,46,47,48]. However, Mager et al. [46] reported that the sub-optimal level of vitamin D found at diagnosis was resolved in half of the coeliac population after 1 year on a GFD. Caruso et al. [45] also observed a normalization of vitamin D and calcium levels after 1–2 years on a GFD.

As regards vitamin K, Mager et al. [46] reported a complete resolution of the deficiency observed at diagnosis after 1 year on a GFD.

### 3.3. Nutritional Profile of Gluten-Containing and GF Food Products

It is known that adherence to a GFD allows the remission of symptoms, normalization of serum antibodies, and intestinal mucosal recovery [49,50]. Thus, the nutritional status of CD patients on a GFD is likely due to the nutritional quality of GF products and to the food choices of CD patients. Population studies have highlighted that the nutritional status of CD subjects following a GFD is not adequate. Hence, it is pivotal to understand the contribution of GF wheat-substitutes to unbalanced nutrient intakes.

Bread and bakery products are traditionally based on flour derived from the cereal wheat, which is, together with other temperate cultivated cereals (e.g., barley and rye), an excellent carrier of macro- and micro-nutrients [51]. Bread contains, for instance, a considerable amount of carbohydrates (≅42.71–51.88 g/100 g) and proteins (≅8.50–12.45 g/100 g bread), and is an important source of micro-nutrients, such as the minerals calcium, iron, zinc, magnesium, phosphorus, potassium, and some B vitamins, including folates [52]. The fat content of bread is low (≅2.15–4.53 g/100 g) [52], and DF content is variable, with a slice of wheat bread (29 g) delivering about 1.2 g of DF [52] and a similar slice of whole grain bread delivering 3.0–4.5 g [53].

GF flours used to formulate GF cereal products are, on the other hand, deficient or poor in some macro- or micro-nutrients. Rice and corn, for instance, which are among the most frequently used raw materials in formulation of GF cereal products, are poor in protein, DF and folate content [54]. Moreover, the need to add to GF formulations surface-active ingredients like starches, and/or proteinaceous and fatty ingredients like dairy and egg proteins, as well as hydrocolloids and gums, to counteract the absence of gluten, poses nutritional problems [55].

When starch is combined with water, at a temperature ranging between 60 and 80 °C, bread volume increases thanks to gelatinization. However, from a nutritional point of view, the more starch is gelatinized, the more it is hydrolysable by α-amylase, and that implies an increase of the food product glycaemic index (GI) [53,55,56]. Hence, GF products formulated with corn and rice starch have a high GI [56,57,58], and their intake may increase the risk of developing metabolic syndromes in coeliac people [58,59,60], as shown by epidemiological studies [59,61,62,63]. The addition of microencapsulated high-fat powder and low-fat dairy powders has also been shown to contribute to making GF foods more palatable, but an increase in the caloric profile has emerged [56]. Moreover, GF products are generally non-fortified and hence do not contain the same level of micro-nutrients than gluten-containing bread.

Over the last five years, some research groups worldwide have undertaken surveys on the nutritional profile of GF food products available on the market based on their labels (Table 2). The collected data provide a picture of the nutritional quality of GF food products available on the market in Australia, Austria, Brazil, Canada, Chile, Italy, Spain, United Kingdom and USA.

**Figure 3:**
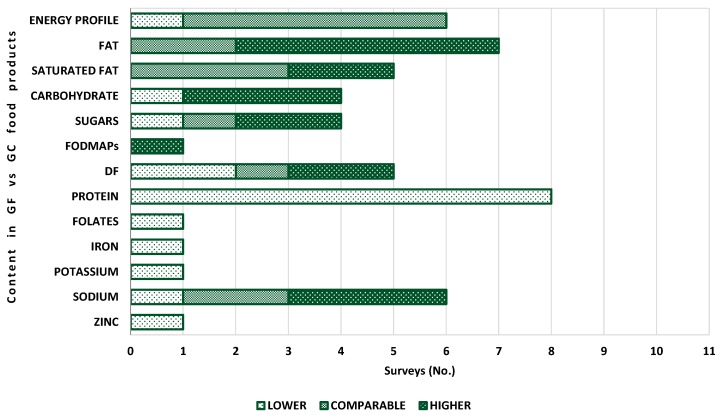
Comparison between GF food products and GC equivalents in terms of energy and nutrient content, as emerged from surveys.

The above mentioned food surveys have shown that there is not an unequivocal nutritional profile for GF food products worldwide. Differences from country to country, from brand to brand, and among food categories have been asserted. Differences between GF products and their equivalents with gluten have also been found [64,65,66,67,68,69,70,71,72,73,74]. Figure 3 displays similarities and differences between GF food products and GC equivalents in terms of macro- and micro-nutrient content, as emerged from the food surveys considered in this paper.

As regards the energy profile, five surveys reported that GF food products exhibit values comparable to GC equivalents [65,67,68,69,72], while one survey found for GF bakery products a lower content [66]. Differences among food categories were found by Nascimento et al. [64]: Comparable values were reported for GF bread and pasta and lower values for cookies, snacks and breakfast cereals. Cornicelli et al. [74] found that the energy content was comparable, except for bread and pasta. The former showed a lower energy content and the latter a higher.

As regards fat, five surveys highlighted a higher content [65,66,69,71,72]. In detail, Miranda et al. [66] found that fat content in GF bread was twice as high (*p* = 0.001) than in standard bread; they also reported that bakery products were higher in cholesterol. Fry et al. [71] observed that GF bread and flours were the food category with the highest fat content, and, in particular, commercial GF foods had a higher fat level than prescribed GF products. Missbach et al. [68] and Estévez et al. [70] found comparable fat content in GF products.

Saturated fat was reported higher in two surveys [66,74] and comparable in three surveys [65,68,72]. Wu et al. [67] found for bread, breakfast cereals and pasta, comparable values between GF and GC products, in agreement with Kulai and Rashid [65] and Allen and Orfila [72], whereas saturated fat was lower in GF cakes and cake mixes.

A higher carbohydrate content was found in GF foods by Kulai and Rashid [65], Mazzeo et al. [69] and Cornicelli et al. [74], while Allen and Orfila [72] observed a lower level in white bread. As regards sugars, two surveys reported a higher content in GF food products [69,71], one comparable [68], and one lower [72]. According to Fry et al. [71], the GF food categories richer in sugar were bread and flours. Moreover, prescribed GF foods showed a generally higher sugar content. Wu et al. [67] found a higher total sugar level (by 8.1 g/100 g, *p* < 0.001) in GF cake mixes and cakes, while pasta, bread and breakfast cereals had comparable values. Chumpitazi et al. [73] found in three GF foods a FODMAP content higher than GC equivalents.

As regards DF content in GF food products, different findings were reported. Kulai and Rashid [65] and Miranda et al. [66] claimed a lower content. Missbach et al. [68] obtained comparable DF level, whereas Wu et al. [67] and Estévez et al. [70] found higher levels. The most recent food surveys by Fry et al. [71], Cornicelli et al. [74] and Allen and Orfila [72] observed different trends, depending on the food category: GF bread was richer in DF compared to GC bread, whereas pasta was poor in DF. This means that efforts have been made to improve some GF food categories. This is important from a nutritional point of view, as DF plays a pivotal role in aiding digestive function, regulating intestinal function, improving the glycaemic response and lowering blood cholesterol.

A complete agreement among surveys was found for protein content: Lower values were detected in GF food products. Bread available on the Spanish market had 30% less protein than its equivalent (*p* < 0.001) [66]. Wu et al. [67] also observed for GF bread, as well as for breakfast cereals and pasta, lower average protein levels, likely due to the ingredients used (e.g., maize starch, white rice flour, potato starch or tapioca starch), which are carbohydrate rich but protein poor. The protein content of GF products available on the Austrian market [68] was more than two times lower in 57% of all food categories (flours/bake mixes, bread and bakery products, pasta and cereal-based products and snacks): 5.8 ± 3.7 g/100 g in GF products versus 8.6 ± 2.9 g/100 g in standard food products. The UK market, investigated by Fry et al. [71], offered GF products with a consistently lower protein content than their GC equivalents, with differences among food categories: 6.2 g lower in GF pizza bases vs. 1.1 g lower in GF biscuits. These data were confirmed by the survey of Allen and Orfila [72], who observed that protein content was significantly lower in white and brown bread (*p* < 0.001) and pasta (*p* < 0.001) when compared to standard products. In the Italian market, protein levels were lower in every food category, with rusks and bread substitutes having the greatest difference [74].

Few data are currently available regarding the vitamin content of GF products, despite nutritional deficiencies emerging from analysis of the nutritional status of CD patients on a GFD. Folates have been investigated by Kulai and Rashid [65], while nicotinic acid, thiamin, folic acid and riboflavin have been detected by Allen and Orfila [72].

Alongside vitamins, few data were collected on mineral content. Iron, potassium and zinc content in GF food products were lower than in GC counterparts. Missbach et al. [68] found that potassium content was significantly lower in GF snacks than in the equivalent products containing gluten (190.4 ± 160 mg/100 g compared to 247.5 ± 130 mg/100 g), whereas no significant difference was observed for bread and bakery products.

Special attention was paid to sodium content. Apart from Missbach et al. [68], who observed a low sodium content in 65% of the studied GF products (<120 mg/100 g), with GF bread and bakery products having a lower sodium content (388.4 ± 206.4 mg/100 g) than in the GC equivalents (581 ± 290.3 mg/ 100 g), a generally higher content in sodium was claimed [66,69,71]. Wu et al. [67] and Allen and Orfila [72] asserted a comparable sodium content.

From the above mentioned considerations, it is apparent that vitamin and mineral content in GF food products should be investigated in order to evaluate the necessity for fortification of GF products. The use of alternative ingredients, such as pseudocereals and legumes, should be also considered in order to improve the protein profile of GF products. A reduction of fat, carbohydrate, sugars and sodium should become a priority for food technologists.

### 3.4. GFD Adherence, Cardiovascular Disease and Metabolic Syndrome

It is well known that an excessive intake of fat, sugars and sodium contributes to the onset of cardiovascular disease and metabolic syndrome (MS), both of which are major public health issues in the Western countries. Hence, defining the effect of any diet (diets for medical purposes included) on the risk for cardiovascular disease and MS is of utmost importance.

The effect of a GFD on the onset of cardiovascular disease and metabolic syndrome has been determined by population studies on CD patients. Parameters such as body mass index (BMI), waist circumference, low density lipoprotein (LDL) cholesterol, triglycerides, blood pressure and insulin resistance have been used to evaluate the cardiovascular risk in coeliac patients adhering to a GFD. Food records have been used to evaluate the adherence to the GFD, and clinical records have sometimes been used to assess the BMI and/or other clinical parameters at diagnosis.

As regards the overweight and obesity status, an increase of BMI in CD children after adhering to a GFD has been commonly observed [75,76,77,78,79,80,81,82,83]. In particular, Więch et al. [84] calculated weight and body composition components in CD children and observed a greater increase in weight, BMI and fat mass in children on a GFD compared to those who did not follow the recommended diet. Kabbani et al. [85] found in a cohort of 679 CD adults (>18 years) that 15.8% of subjects moved from normal/low BMI into an overweight BMI, and 22% of patients that were overweight at diagnosis gained weight [85]. In contrast, Ukkola et al. [86] found that 69% of underweight patients at diagnosis gained weight while on a GFD, while 18% of overweight and 42% of obese patients lost weight. More recently, Barone et al. [39] reported no changes to overweight or obese status after following a GFD in 82% of 39 CD patients, which were showing normal or overweight BMI at diagnosis. Besides an obesogenic behavior observed in children and their families in response to initiation of gluten-free diet [87], among the causes for weight gain of subjects on a GFD, the “compensatory hypothesis”, that is, the normalization of caloric balance due to the recovery of mucosal functionality, has been proposed [88]. However, other factors, such as the higher fat and protein content of GF food, products with respect to their GC counterparts, and the high glycaemic index thereof [75] could contribute to weight gain. Moreover, it should be noted that GF food products are perceived as safer than GC foods; hence, there is a trend in CD subjects to eat more. More recently, Papastamataki et al. [89] observed a different secretion pattern of gut–brain axis hormones in coeliac children compared with healthy controls, which may contribute to weight gain.

As regards the investigation of MS risk, Tortora et al. [90] found that 2% of CD patients met the criteria for MS at diagnosis, but that this percentage increased to 29.5% after 12 months on a GFD (*p* < 0.01). Ciccone et al. also observed that following a GFD increased the risk of developing MS in CD patients from 3.24% at diagnosis to 14.59% after a GFD (*p* < 0.0001) [91]. In contrast, Kabbani et al. [92] found that the prevalence of metabolic syndrome was lower among CD patients on a GDF diet than in controls and the general population. Zifman et al. [93] found in a pediatric CD cohort that one year adherence to a GFD was not associated with increased CVD risk factors; however, the long term effect of a GFD should be further investigated. A systematic review by Potter et al. [94] reports that a GFD alters some cardiovascular risk factors in CD patients, although further studies are warranted to evaluate the overall effect. Due to the increased popularity of GFD among the population not affected by gluten-related disorders [95,96], the effect of GFD on cardiovascular risk was also evaluated in healthy subjects. Kim et al. [96] reported that following GFD did not significantly affect MS prevalence and cardiovascular risk in healthy subjects. Ehteshami et al. [97] evaluated the effect of GFD on subjects not affected by CD but diagnosed with MS, and found that short-term GFD reduced waist circumference and improved glycemic control and triglyceride levels.

### 3.5. Improving GFD for Gut Microbiota Recovery

Diet has also been found to play a pivotal role in the composition and function of the gut’s microbiota, which in turn influences the host’s health via multiple routes [98]. The composition of the gut microbiota changes from birth to adulthood. In adults it is generally stable; however, variations can occur in response to diet change, gastrointestinal disease and antibiotic treatment.

Two main mechanisms have been proposed to explain the impact of diet on microbiota composition. First, microbial species compete for substrates of dietary origin, and differences in the ability to use substrates explain why certain microbial species favored over others in case of greater quantities of specific substrates. In addition, gut environment characteristics, namely pH, bile salt concentrations and micronutrient concentrations, which are deeply affected by diet, may influence microbial growth [98].

Observational studies [99,100,101] of subjects with CD have shown alterations in the gut microbiota composition compared to healthy subjects, and this dysbiosis has been found to persist in coeliac patients in remission and on a GFD.

It emerged that bacteria generally recognized as healthy (*Bifidobacterium*, *B. longum* and *Lactobacillus* spp.) decrease in number in subjects on a GFD, while potentially unhealthy bacteria, such as *Enterobacteriaceae* spp. increase in number.

Observed changes in gut composition are likely due to the reduced intake of polysaccharide associated with a GFD [102]. These macronutrients commonly reach the colon’s distal part partially undigested and provide energy to commensal species of the gut microbiota. So, in case of a reduced polysaccharide intake, microbial species compete for substrates and opportunistic pathogens overgrow. This explains why De Palma et al. [103] found a decrease in *Bifidobacterium* spp., *Clostridium lituseburense* group, *Fecalibacterium prausnitzii*, *Lactobacillus* spp. and *Bifidobacterium longum*, and an increase in *Escherichia coli*, *Enterobacteriaceae* and *Bifidobacterium angulatum*, in subjects on a one month GFD.

As a consequence of the overgrowth of opportunistic species, the host defenses against infection and chronic inflammation might weaken.

It should also be considered that short chain fatty acids (SCFAs) are obtained from polysaccharide fermentation. SCFAs generate in gut conditions hostile to enterobacteria. During periods of lower polysaccharide levels, fewer SCFAs form. Nevertheless, DF might promote a greater short-chain fatty acid concentration, thus fostering microbial colonization [102].

Hence, an improved GFD may contribute to increasing the count of beneficial bacteria and reducing the count of harmful microbial species, thus enabling GFD treated patients a recovery of the gut ecosystem. However, the possibility to improve the health status of CD patients by managing the microbiota should be investigated. Moreover, since the intake of dietary components, such as proteins, fibers, micronutrients, can influence and shape the functional microbiome [104], a tailored diet could be helpful in restoring a balanced gut microbiota.

### 3.6. Limitations of the Review

Possible limitations of this study must be highlighted. The literature search was conducted on the Scopus and PubMed databases only. Other databases were not accessible to the authors. The literature search was limited to the last five years, which is a limitation but also a strength. Very up-to-date data were analyzed and considered. The aim of the work was, in fact, to evaluate the nutritional composition of GF foods currently available on the market and currently consumed by coeliac people, and to examine their effects on the nutritional status of CD patients.

As regards composition data of GF food products, only a limited number of food surveys were available. The surveys were performed in different countries: Canada, Spain, Australia, Austria, Italy, Chile, UK, USA. Hence, the obtained compositional data might be affected by differences from brand to brand and the dietary preferences of each country. Moreover, results might be also affected by the number of sampled products and by categories therein. Finally, the cut-off for gluten content in GF products is not harmonized [105]; thus, some products falling under the “gluten-free” category in some countries do not in others.

As regards the effect of a GDF on patient nutritional status, few studies are available and they occasionally include a limited number of subjects. Additionally, few studies are available on the analysis of GFD effects on MS and gut microbiota.

## 4. Conclusions

The most recent surveys on the nutritional quality of GF food products currently available on the market show key inadequacies—a low protein content and a high fat and salt content—compared to their equivalent gluten-containing products. However, an interesting trend towards some improvements has emerged: More adequate levels of fiber and sugar than in the past have been reported in the surveys of the last two years. Further surveys are nevertheless required to investigate the micronutrient content of GF food products, which has been so far overlooked.

Population studies highlighted CD patients’ inadequate intake of fats, proteins, sodium and vitamins, while adhering to a GFD. However, studies comparing nutrient levels before and after the start of the GFD should be performed, in order to better understand the effect of the GFD on the nutritional status of CD patients. The risk of co-morbidities should also be monitored.

To conclude, a further effort by food technologists, nutritionists, the food industry and its regulators is required to improve the nutritional quality of GF food products and hence the health of CD patients adhering to the GFD. The use of alternative ingredients and technologies should be exploited in order to increase the content of micronutrients and DF in GF products.

## Figures and Tables

**Table 1:** Literature database queries.

PubMed	No. of Documents Found	Scopus	No. of Documents Found
Search		Search	
(((coeliac disease OR celiac disease)) AND gluten-free diet) AND nutrition	316	(TITLE-ABS-KEY (coeliac AND disease OR celiac AND disease) AND TITLE-ABS-KEY (gluten-free AND diet) AND TITLE-ABS-KEY (nutrition))	220
((gluten-free food OR gluten-free food product)) AND ((nutritional profile OR nutritional composition))	61	(TITLE-ABS-KEY (gluten-free AND food OR gluten-free AND food AND product) AND TITLE-ABS-KEY (nutritional AND profile OR nutritional AND composition))	78
(coeliac disease OR celiac disease) AND (nutritional inadequacies)	3	(TITLE-ABS-KEY (“coeliac disease OR celiac disease”) AND TITLE-ABS-KEY (“nutritional inadequacies”))	53
(((“coeliac disease” OR “celiac disease”)) AND gluten-free diet) AND deficiency	112	TITLE-ABS-KEY (coeliac AND disease OR celiac AND disease) AND TITLE-ABS-KEY (gluten-free AND diet) AND TITLE-ABS-KEY (deficiency)	328
((“coeliac disease” OR “celiac disease”)) AND malnutrition	129	TITLE-ABS-KEY (coeliac AND disease OR celiac AND disease) AND TITLE-ABS-KEY (malnutrition)	157
(((coeliac disease OR celiac disease)) AND gluten-free diet) AND fat intake	14	(TITLE-ABS-KEY (“gluten-free diet”) AND TITLE-ABS-KEY (“fat intake”))	28
(((coeliac disease OR celiac disease)) AND gluten-free diet) AND carbohydrate intake	19	(TITLE-ABS-KEY (“gluten-free diet”) AND TITLE-ABS-KEY (“carbohydrate intake”))	12
(((coeliac disease OR celiac disease)) AND gluten-free diet) AND protein intake	45	(TITLE-ABS-KEY (“gluten-free diet”) AND TITLE-ABS-KEY (“protein intake”))	44
((“coeliac disease” OR “celiac disease”)) AND micronutrients	104	TITLE-ABS-KEY(coeliac disease OR celiac disease) AND TITLE-ABS-KEY(micronutrients)	61
((coeliac disease OR celiac disease)) AND vitamin	195	TITLE-ABS-KEY(coeliac disease OR celiac disease) AND TITLE-ABS-KEY(vitamins)	397
((“coeliac disease” OR “celiac disease”)) AND minerals	22	TITLE-ABS-KEY(coeliac disease OR celiac disease) AND TITLE-ABS-KEY(minerals)	169
(((coeliac disease OR celiac disease)) AND gluten-free diet) AND obesity	22	(TITLE-ABS-KEY (gluten-free diet) AND TITLE-ABS-KEY (obesity))	99
(((coeliac disease OR celiac disease)) AND gluten-free diet) AND microbiota	60	(TITLE-ABS-KEY (gluten-free diet) AND TITLE-ABS-KEY (microbiota))	107
(((coeliac disease OR celiac disease)) AND gluten-free diet) AND metabolic syndrome	17	(TITLE-ABS-KEY (gluten-free diet) AND TITLE-ABS-KEY (metabolic syndrome))	24
(((coeliac disease OR celiac disease)) AND gluten-free diet) AND (glycaemic index OR glycemic index)	12	(TITLE-ABS-KEY (gluten-free diet) AND TITLE-ABS-KEY (glycaemic index OR glycemic index))	29
((coeliac disease OR celiac disease)) AND (co-morbidity OR co-morbidities)	136	(TITLE-ABS-KEY (coeliac disease OR celiac disease) AND TITLE-ABS-KEY (co-morbidity OR co-morbidities))	33
((coeliac disease OR celiac disease)) AND (gluten-free diet compliance)	161	(TITLE-ABS-KEY (coeliac disease OR celiac disease) AND TITLE-ABS-KEY (“gluten-free diet compliance”))	4
((coeliac disease OR celiac disease)) AND ((cardiovascular diseases OR cardio-vascular diseases))	382	(TITLE-ABS-KEY(coeliac disease OR celiac disease) AND TITLE-ABS-KEY(“cardiovascular diseases” OR “cardio-vascular diseases”))	37
((coeliac disease OR celiac disease)) AND (weight gain)	40	(TITLE-ABS-KEY (coeliac disease OR celiac disease) AND TITLE-ABS-KEY (“weight gain”))	38
**TOTAL**	**1895**		**1918**

**Table 2:** Surveys on the nutritional profile of GF food products available on the worldwide market.

Reference	Publication Year	Geographical Area	No. Products		Food Category	Nutritional Profile of GF Food Products
Nascimento et al. [64]	2013	Brazil	168 (GF)	162 (GC)	cookies	Higher energy profile Higher protein, saturated fat and sodium content
					bread and pasta	Lower protein and DF content
					snacks	Lower energy profile Lower total fat, saturated fat and sodium content Higher protein and DF
					breakfast cereals	Lower energy profile Higher sodium content
Kulai and Rashid [65]	2014	Canada	71 (GF)	60 (GC)	GF food products	Energy profile comparable to GC food products;
					bread	Higher fat content, two-fold No differences in saturated fat content Lower protein content
					pasta	Higher mean carbohydrate content Low in dietary fibre, iron, folates
					breakfast cereals and cake mixes	No significant difference
Miranda et al. [66]	2014	Spain	206 (GF)	289 (GC)	bread	Higher fat content, especially saturated fat Lower protein content More salt Less fibre
					pasta	Nutrient profile similar to *bread*
					bakery products	Lower energy, protein and carbohydrate content Higher sodium and cholesterol content
						Differences among brands
Wu et al. [67]	2015	Australia				Similar nutritional profile between GF and GC food products
					pasta, bread, breakfast cereals	Lower protein content Similar total energy, sodium, saturated fats, total sugars
					bread	High mean DF content
					cereal bars, cake mixes, sweet biscuits	High content of sugar, saturated fats and salt
					cake mixes, cakes	Low saturated fat levels High total sugar level Total energy similar to GC
Missbach et al. [68]	2015	Austria	63 (GF)	126 (GC)	-	Energy content, carbohydrates, total fats, saturated fatty acids, fibre and sugar did not differ between GF and GC products Protein content more than two-folds lower in 57% of all GF food categories Sodium content lower in GF products Potassium content was overall significantly lower in GF food products
					pasta	Zinc content significantly lower in GF pasta products
Mazzeo et al. [69]	2015	Italy	60 (GF)		sweet products	High fat and sugar content
					brioches	High content of salt
					bread, pizza, snack, flours	High available carbohydrate and sugar content
Estévez et al. [70]	2016	Chile	19 (GF)	34 (GC)	bread	Low protein content High DF content
						Fat content similar to GC products.
Fry et al. [71]	2018	United Kingdom	679 (GF)	1045 (GC)	-	Lack of a pattern in the comparison of overall nutritional quality of GF dietary foods
					biscuits, crackers, white and brown bread, breakfast cereals, white and wholegrain flour, pizza bases, wholegrain and white pasta	Low protein content
					commercial white bread, breakfast cereals, wholegrain pasta	High and medium fat and saturated fats
					prescribed breakfast cereals, crackers, biscuits	High and medium salt content
					brown and white bread, white and wholegrain flour, pizza bases, crackers, biscuits	Higher sugar content in prescribed GF food products than commercial
					wholegrain flours, white pasta	Very high salt content
					white and brown bread	High DF content
					breakfast cereals, white and wholegrain pasta	Low DF content
Allen and Orfila [72]	2018	United Kingdom	49 (GF)	61 (GC)	-	Average total energy, saturated fat, and salt values were similar between GF and GC products
					brown bread, pasta	Lower levels of sugar
					white, brown, seeded bread	Higher fat content
					white, brown, seeded bread, pasta	Lower carbohydrate intake per portion
					white, brown, seeded bread, pasta	Lower protein content
					white, brown, seeded bread	Higher DF content
					GF pasta	Significantly lower DF content
					bread	Only 28% of GF breads were fortified with calcium carbonate and iron Only 5% of the total GF breads were fortified with all four fortification minerals, in addition to folic acid and riboflavin.
Chumpitazi et al. [73]	2018	USA	3 (GF)		-	excessive FODMAP content
Cornicelli et al. [74]	2018	Italy	235 (GF)	349 (GC)	-	Overall, energy content was not different to that of regular equivalents. Two exceptions: Lower content in bread and higher in pasta
					all GF food categories, but especially bread and rusks	Lower protein content
					-	Fat content was not different. GF biscuits and pasta have the highest content of saturated fats;
					GF biscuits, rusks, pasta and bread substitutes	Higher content of carbohydrates
					GF biscuits, bread substitutes, pasta	Lower DF content
					GF bread	Higher DF content
					GF pasta and rusks	Higher salt content
					GF biscuits	Lower salt content
